# Infiltration of Proinflammatory M1 Macrophages into the Outer Retina Precedes Damage in a Mouse Model of Age-Related Macular Degeneration

**DOI:** 10.1155/2013/503725

**Published:** 2013-03-07

**Authors:** Fernando Cruz-Guilloty, Ali M. Saeed, Jose J. Echegaray, Stephanie Duffort, Asha Ballmick, Yaohong Tan, Michel Betancourt, Eduardo Viteri, Ghansham C. Ramkhellawan, Eric Ewald, William Feuer, DeQiang Huang, Rong Wen, Li Hong, Hua Wang, James M. Laird, Abdoulaye Sene, Rajendra S. Apte, Robert G. Salomon, Joe G. Hollyfield, Victor L. Perez

**Affiliations:** ^1^Department of Ophthalmology, Bascom Palmer Eye Institute, University of Miami Miller School of Medicine, Miami, FL 33136, USA; ^2^Department of Microbiology and Immunology, University of Miami Miller School of Medicine, Miami, FL 33136, USA; ^3^Department of Chemistry, Case Western Reserve University, Cleveland, OH 44106, USA; ^4^Department of Ophthalmology and Visual Sciences, Washington University School of Medicine, St. Louis, MO 63110, USA; ^5^Department of Ophthalmology, Cole Eye Institute, Cleveland Clinic Lerner College of Medicine, Cleveland, OH 44195, USA; ^6^Ophthalmology, Immunology & Microbiology, University of Miami Miller School of Medicine, Bascom Palmer Eye Institute, Miami, FL 33136, USA

## Abstract

Age-related macular degeneration (AMD) is a major cause of blindness in the developed world. Oxidative stress and inflammation are implicated in AMD, but precise mechanisms remain poorly defined. Carboxyethylpyrrole (CEP) is an AMD-associated lipid peroxidation product. We previously demonstrated that mice immunized with CEP-modified albumin developed AMD-like degenerative changes in the outer retina. Here, we examined the kinetics of lesion development in immunized mice and the presence of macrophages within the interphotoreceptor matrix (IPM), between the retinal pigment epithelium and photoreceptor outer segments. We observed a significant and time-dependent increase in the number of macrophages in immunized mice relative to young age-matched controls prior to overt pathology. These changes were more pronounced in BALB/c mice than in C57BL/6 mice. Importantly, IPM-infiltrating macrophages were polarized toward the M1 phenotype but only in immunized mice. Moreover, when Ccr2-deficient mice were immunized, macrophages were not present in the IPM and no retinal lesions were observed, suggesting a deleterious role for these cells in our model. This work provides mechanistic evidence linking immune responses against oxidative damage with the presence of proinflammatory macrophages at sites of future AMD and experimentally demonstrates that manipulating immunity may be a target for modulating the development of AMD.

## 1. Introduction

Age-related macular degeneration (AMD) is the most common cause of legal blindness in the elderly population of developed countries with over 300,000 newly diagnosed patients per year in Europe and North America [1, 2]. It is widely believed that AMD starts with the insidious, slowly progressing “dry” form (dry AMD) and can later develop into the more severe “wet” AMD, which advances very rapidly and is characterized by abnormal development of blood vessels, a process called choroidal neovascularization (CNV) [3, 4] that affects the macular region of the retina and leads to loss of central vision. In turn, dry AMD without CNV can proceed to focal loss of the retinal pigment epithelium (RPE), termed geographic atrophy (GA), which is accompanied by loss of vision over these slowly expanding areas of RPE atrophy [5]. RPE loss or dysfunction renders the surrounding tissue vulnerable to damage by reactive oxygen species and loss of photoreceptor density ensues [6]. It has been recognized that accumulation of debris below the RPE in the macula, also known as drusen, is a risk factor for AMD. Identification of complement factor proteins in drusen from AMD eyes [7], coupled with genetic variation in several complement factor genes in AMD patients [8–13], collectively implicates inflammation as an important component in the pathophysiology of this disease [14, 15]. For instance, it was recently demonstrated that complement factor H (CFH) serves a protective role in AMD by binding and inhibiting the inflammatory effects of the lipid peroxidation product malondialdehyde (MDA) [16]. Therefore, dissecting the initiating events of outer retinal inflammation during the early stages of AMD can lead to a better understanding of its pathogenesis. Such findings will enable the development of innovative immunotherapies that can prevent inflammatory damage to retinal tissue and loss of vision in a disease process that is estimated to increase in prevalence by 50% by the year 2020 [17].

In the past decade, several novel therapeutic agents have been identified as effective drugs to treat wet AMD, which delay new blood vessel formation and improve vision [18]. However, there is no effective treatment for dry AMD to date. In the pursuit of identifying the signals from the outer retina that initiate inflammation and possibly involve the immune system in AMD pathogenesis, we have evaluated immune responses to carboxyethylpyrrole (CEP), a protein modification that forms from an oxidation fragment of docosahexaenoic acid (DHA) [19], the most oxidizable of all long chain polyunsaturated fatty acids. Studies have shown that AMD donor eyes contain more CEP-modified proteins in the outer retina and drusen than in age-matched controls [7]. CEP-modified proteins and CEP autoantibodies are also more abundant in AMD plasma than in control samples [19, 20].

Since DHA is abundant in the outer retina [21], where the amalgamation of high oxygen tension and light provides an environment suitable for oxidative damage, our lab has previously developed a murine model in which mice immunized with CEP-modified mouse serum albumin (CEP-MSA) develop a CEP-specific immune response which correlates with dry AMD-like pathology when compared to age-matched controls, including CEP autoantibodies, complement deposition in the Bruch's membrane, and RPE damage [22, 23]. To our knowledge, this is the first immune-mediated mouse model of dry AMD in genetically unmanipulated animals and stems directly from observations in human patients.

Another component of the immune system that has been implicated in AMD is the macrophage lineage [24, 25], although the specific role of these innate immune cells at different stages of AMD disease progression is still controversial. Within the retina, there are two sources of macrophages: (1) microglia, bone marrow-derived resident macrophages that are recruited to neural tissue during retinal development and provide immunosurveillance in the inner retina, and (2) circulating monocytes that can be recruited from the blood vessels to sites of inflammation when needed by specific chemokines and cytokines. Independent of the source, these cells can undergo a diverse program of differentiation depending on the microenvironment that ultimately dictates their effector functions [26, 27]. Macrophages activated in the presence of interferon-gamma (IFN-*γ*) become proinflammatory M1 macrophages, characterized by their production of tumor necrosis factor alpha (TNF-*α*) and interleukin-12 (IL-12) and are associated with tissue damage. On the other hand, macrophages activated in the presence of IL-4 differentiate into M2-type, marked by production of the immunosuppressive cytokine IL-10 and involved in tissue remodeling. The CEP-MSA-induced changes in the outer retina that provide a model for AMD afford the unique opportunity to directly test the role of these cells in the disease process.

Our current study aims to characterize both the magnitude and kinetics of development of retinal lesions and macrophage involvement in the BALB/c and C57BL/6 (B6) mouse strains at various intervals postimmunization (p.i.) in young mice compared to age-matched controls, before extensive retinal lesions are observed. Here we extend our original study [22] by showing that CEP-MSA immunization leads, in aged (old) BALB/c mice, to the end stage cardinal feature of human dry AMD: loss of photoreceptor cells. This major damage is the result of a low-grade but significant inflammatory response in the retina prior to overt tissue damage, which can be quantified in young mice. We have identified M1 macrophages localized to the interphotoreceptor matrix (IPM) surrounding the photoreceptor outer segments in close proximity to the RPE. These changes occur in both BALB/c and B6 strains, but the kinetics are different; BALB/c mice are more susceptible at a younger age. We also detected elevated levels of the monocyte chemoattractant Ccl2 in the retinas of CEP-immunized mice. Moreover, *Ccr*2^−/−^ B6 mice immunized with CEP-MSA lack macrophage recruitment, and retinal lesion development is reduced or prevented. Since AMD is an age-related disease, defining the progression of inflammatory cell recruitment and development of AMD-like lesions at earlier stages of the disease is essential in order to map the character and timing of immune mechanisms that take place in our model and correlate with development of pathology. This work clarifies a long-standing question by defining a clear mechanistic path that explains the role of inflammation in AMD: M1 macrophages are key factors in dry AMD pathogenesis. We also provide experimental demonstration for the idea that regulation of immune responses (in this case, inhibition of macrophage recruitment) can be a target of therapy to prevent the development of AMD.

## 2. Results

### 2.1. Magnitude and Tempo of Lesion Development and Inflammatory Cell Recruitment

**Figure 1:**
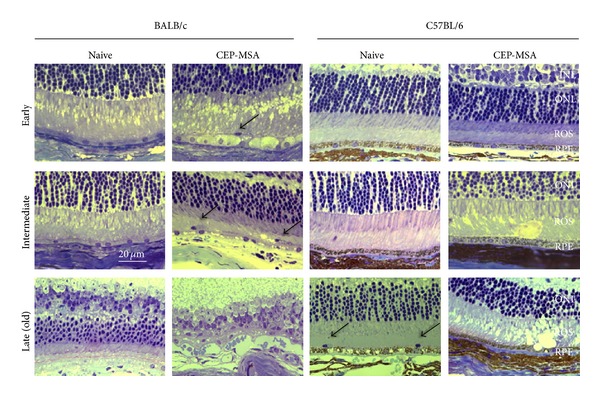
Immunization with CEP-MSA leads to overt retinal degeneration in aged mice that resembles geographic atrophy with loss of photoreceptor cells, particularly in the BALB/c background. Histology of CEP-MSA versus naïve BALB/c or C57BL/6 eyes at different time points postimmunization. The RPE is located at the lower part of each image. The dark arrows show macrophage-like cells. CEP-MSA BALB/c mice show strong pathology since the early time points, starting with swollen RPE cells and leading to massive geographic atrophy at the late time point, including complete loss of the photoreceptor cells. The kinetics of pathology in CEP-MSA B6 mice is slower, but eventually there are focal lesions of the RPE such as vesiculation, as previously reported [21]. Representative images are shown (from 3–5 mice per group per experiment; two or three independent experiments were performed for each strain). INL: inner nuclear layer; ONL: outer nuclear layer; ROS: rod outer segment; RPE: retinal pigment epithelium. Images were obtained using a 63x oil objective, and the scale marker represents a 20 *μ*m length.

We have previously described AMD-like lesions in wild-type (WT) B6 mice immunized with CEP-MSA [22]. We now describe in detail lesions in CEP-MSA-immunized mice on the BALB/c background, which are inherently albino. Importantly, but also technically challenging, the severity of lesions increases with time after immunization, and it can take for up to 12–24 months to observe signs of geographic atrophy and photoreceptor cell loss, cardinal features of AMD (Figure 1, Supplemental Figure  S1 available online at http://dx.doi.org/10.1155/2013/503725). The main purpose of the current study was to determine the earliest time at which significant differences between CEP-immunized and control mice could be detected. The reasoning behind this approach is to define the molecular and cellular mechanisms that take place at the initial stages of disease, before there are gross changes to the retina that could alter its function. These early time points could also be critical for therapeutic intervention.

Eyes harvested at 40–90 days p.i. were defined as of early recovery times, those harvested at 100–200 days p.i. were considered of intermediate recovery times, while those obtained after day 200 p.i. were considered of late recovery times. Focal lesions in the RPE and IPM consisting of vacuolization of individual or groups of cells, pyknotic RPE cells, hypertrophic RPE cells, and melanin engulfment by macrophages (only possible in B6 mice), as well as darkly stained nuclei of inflammatory (macrophage-like) cells in the RPE and IPM were counted (refer to Methods for detailed scoring parameters) (Figure 1). While significant damage can be observed in the retina of aged (12–24 months old) control mice (mainly thinning of the photoreceptor layer, Figure 1, Supplementary Figure 1), no GA pathology is seen in mice that did not receive CEP immunization or in younger CEP-immunized mice.

**Figure 2:**
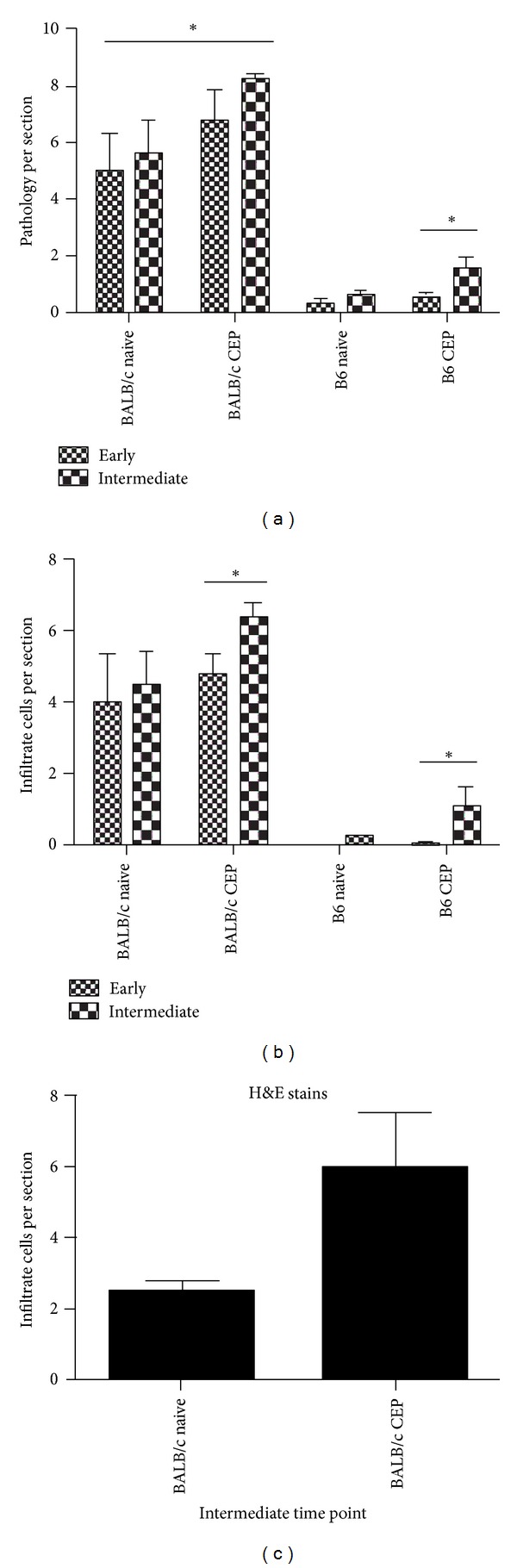
Quantification of retinal pathology and macrophage infiltration in CEP-MSA-immunized mice. (a) Combined pathology score per section (all kinds of lesions and IPM-infiltrating cells). The significant changes due to CEP-MSA immunization occur at the early time point in the BALB/c background, whereas they occur at intermediate time points for B6 mice. (b) CEP-MSA immunization accelerates IPM-infiltrating cell recruitment at early stages of AMD development, prior to major retinal damage. Mean values are shown; error bars represent S.D. (*) denotes statistically significant differences (*P* < 0.05). Data from one representative experiment was used for this analysis; similar results were obtained in additional repeat experiments. (c) Haematoxylin & Eosin (H&E) staining of frozen sections confirmed the accelerated macrophage recruitment associated with CEP-MSA immunization. 5 sections were scored per mouse, with 3 mice per group at the intermediate time point (d 100–200 p.i.). Mean values are shown; error bars represent S.D.

Our data was analyzed with a three-factor analysis of variance; the factors are (1) strain of mice (BALB/c versus B6), (2) immunization status (naïve versus immunized), and (3) time of recovery of tissue (early versus intermediate) (Figure 2). Pathology scores (defined as the summation of retinal lesions and cellular infiltration) were higher in BALB/c CEP-immunized mice at both early and intermediate recovery times but only higher in B6 CEP-immunized mice at the intermediate recovery time (Figure 2(a)). To have a representative and objective pathological score throughout the retina, we focused on the number of IPM-infiltrating cells present in close proximity to the RPE. Using routine histopathology, it is not possible to distinguish different types of monocyte lineage cells present in the outer retina (for example, resident microglia versus macrophages recruited from the circulation). For this reason in the description of the results to follow, we will simply refer to the nonneural cells in the IPM as macrophages, since microglia are distinguished mainly for their location within the central nervous system. Careful examination of the lesions at these early and intermediate recovery times demonstrated that a distinct population of macrophages is present in the IPM compartment near the RPE in these animals. Quantification in plastic sections showed that CEP-immunized BALB/c mice have a significantly higher number of macrophages than age-matched naïve animals harvested at 40–100 days p.i. (*P* = 0.023) as well as those harvested at 100–200 days p.i. (*P* = 0.023) (Figure 2(b)). The same cellular quantification is observed with H&E staining of frozen sections (Figure 2(c)). When comparing IPM macrophages between the two time points, BALB/c mice showed an increase in the number of these cells in the early to intermediate recovery times in both immunized and naïve mice, but the magnitude was significantly higher in immunized mice (*P* = 0.012). While CEP-immunized B6 mice harvested at early recovery times showed no significant differences in IPM macrophages when compared to naïve B6 mice, immunized mice harvested at intermediate time points had more IPM macrophages than age-matched B6 naïve controls (*P* = 0.023). Therefore, the number of monocyte-lineage cells present in the IPM increases over time in CEP-immunized B6 mice to reach significant numbers by the intermediate recovery times.

### 2.2. Enhanced Immune-Mediated AMD-Like Pathology Is Specific to Genetic Background rather than Light Damage

**Figure 3:**
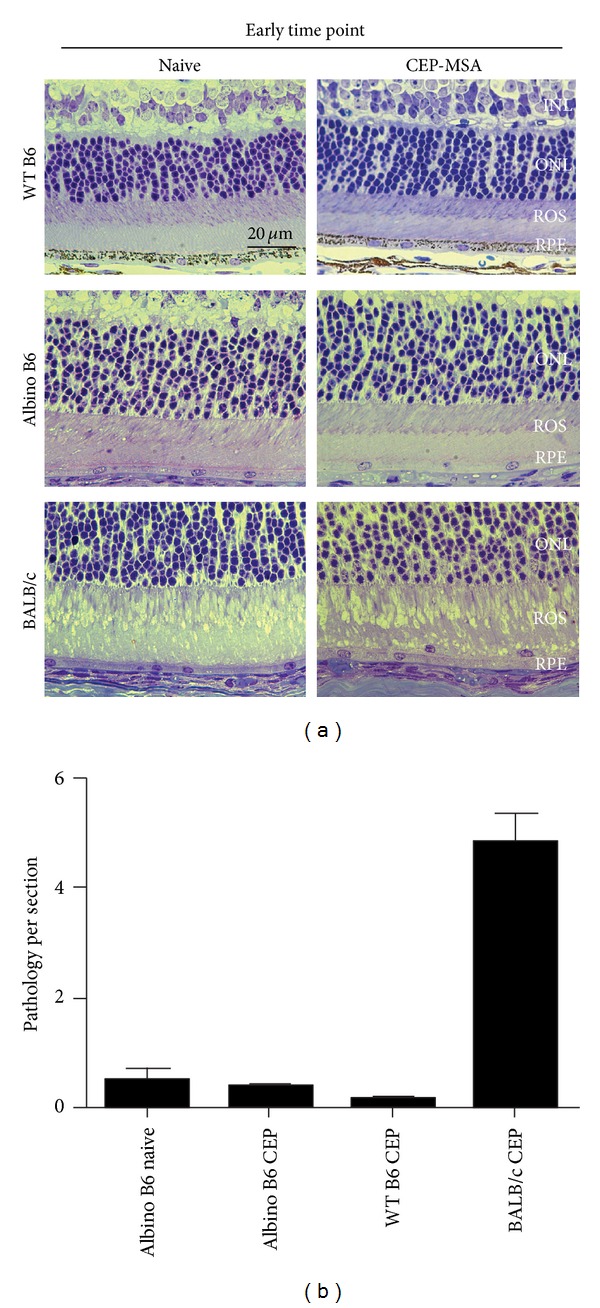
Immune-mediated cellular infiltration and development of outer retinal lesions are specific to genetic background and pigment independent. (a) Histology of CEP-MSA albino B6 mice compared to WT BALB/c and WT B6 at the early time point. Representative images are shown (from 3–5 mice per group per experiment; two or three independent experiments were performed for each strain). INL: inner nuclear layer; ONL: outer nuclear layer; ROS: rod outer segment; RPE: retinal pigment epithelium. Images were obtained using a 63x oil objective, and the scale marker represents a 20 *μ*m length. (b) Quantification shows the lack of pathology at early time points in albino B6 mice, similar to WT B6 and in contrast to WT BALB/c mice. Mean values are shown; error bars represent S.D.

Naïve BALB/c mice contained more macrophages in the IPM than age-matched naïve B6 mice (*P* < 0.01), and immunized BALB/c mice show higher number of IPM macrophages than immunized B6 mice as well (*P* < 0.01). This difference is also evident in the number of RPE lesions, which are higher in BALB/c mice than in B6 mice, regardless of immunization status. To test the possibility that photosensitivity in the BALB/c albino mice [28] could be responsible for the differences in outer retinal lesion development and macrophage presence when compared to B6 mice, we immunized albino B6 mice (B6(Cg)-*Tyr* ^*c*−2*J*^/*J* or *Tyr* ^−/−^ mice, which lack the tyrosinase enzyme) and compared them to age-matched naïve *Tyr* ^−/−^ mice as well as to corresponding BALB/c and WT-B6-immunized and naïve mice at 40–90 days p.i. The results from these comparisons showed that both macrophages in the outer retina and development of retinal lesions observed in *Tyr* ^−/−^ mice are comparable to WT B6 and do not show the enhanced cellular recruitment, RPE hypertrophy, and ROS vacuolization present in BALB/c mice at the early time points, regardless of immunization status (Figure 3(a)). Quantification analysis of pathological changes per section supports these observations (Figure 3(b)). This suggests that the differences in inflammatory cells present in the outer retina and AMD-like pathology observed between the BALB/c and the B6 strains are not due to photosensitivity in the albino mice because of lack of pigmentation, but instead they are specific to genetic background, possibly due to differences in the immune responses against CEP-MSA between the mice or to inherent differences in RPE function and/or local oxidative damage responses in the retina.

### 2.3. Inflammatory Cells Surround the Retina of CEP-MSA-Immunized Mice

Because inflammation associated with macrophages present in the IPM seems to play a role in the retinal pathology observed in CEP-MSA-immunized mice, we performed immunohistochemical (IHC) analysis using cell surface immune markers for the identification of the specific macrophages present in the retina, generally, and to the IPM region, specifically. A large number of CD45+ (a pan-leukocyte marker) cells were observed in the choroid of immunized mice compared to controls (Supplementary Figure 2), indicating that CEP immunization leads to increased ocular inflammation. In terms of specific cell subsets, we observed only a few CD3+ and CD19+ cells in the outer retina, suggesting that both T cells and B cells are largely absent from the site of retinal lesions (data not shown). In contrast, there were substantial numbers of choroidal CD11b+, F4/80+, and CD68+ cells surrounding the retinas of BALB/c CEP-MSA-immunized mice and not of naïve controls (data not shown). Similar findings were observed in B6 immunized animals at late recovery times when lesions are present.

### 2.4. Proinflammatory M1 Macrophages Are Present in the IPM at Early Recovery Times in Mice Immunized with CEP-MSA

**Figure 4:**
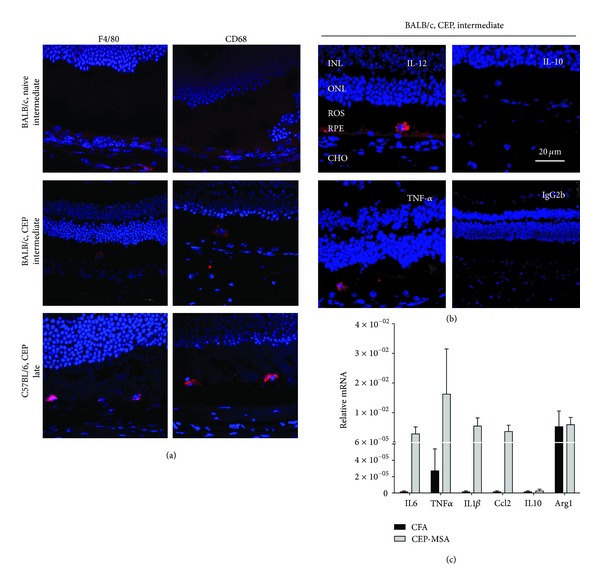
CEP-MSA immunization leads to M1 (TNF-*α*, IL-12 producing) macrophage recruitment and activation in the subretinal space. (a) Frozen sections followed by immunostaining and confocal microscopy. Surface marker stains were used to identify macrophages as the infiltrate cells. F4/80+ and CD68+ cells are absent from the RPE of naive mice, but they are found in CEP-MSA-immunized BALB/c and B6 mice. (b) Intracellular stains for TNF-*α*, IL-12, and IL-10 production were used to determine the phenotype of the activated macrophages observed in CEP-MSA BALB/c mice at the intermediate recovery time. IgG2b was used as isotype control. Representative images are shown (from 3–5 mice per group per experiment; two or three independent experiments were performed for each strain). INL: inner nuclear layer; ONL: outer nuclear layer; ROS: rod outer segment; RPE: retinal pigment epithelium; CHO: choroid. The scale marker represents a 20 *μ*m length. (c) Infiltrating macrophages from B6 mice were isolated by laser microdissection, and RNA was obtained for qPCR analysis of gene expression. Relative mRNA (in arbitrary units) was calculated using the 2^−ΔCt^ method with Actin as the calibrator gene. Transcripts for M1 marker genes (IL-1*β*, TNF, and Ccl2) were detectable in 3 out of 5 CEP-MSA-immunized mice but were not present in CFA age-matched controls (*n* = 4). M2 marker expression did not correlate with CEP immunization: IL-10 was completely absent, whereas the levels of Arg-1 did not increase. Results are representative of at least two independent laser capture experiments.

Immunofluorescence staining identified retinal CD11b+, F4/80+, and CD68+ cells in mice immunized with CEP-MSA, suggesting the potential role of macrophages in the development of disease in this model. We were able to localize a significant number of these macrophages in the RPE and IPM using nuclear counterstaining with DAPI (Figure 4(a), Supplementary Figure 3). This correlates with the location of the macrophages identified by histopathology using toluidine blue and H&E staining that are mostly observed in proximity with vacuolization and lesions of RPE and photoreceptors. In order to determine the specific class and activation status of these macrophages, we performed intracellular stains for Tumor Necrosis Factor-alpha (TNF-*α*) and Interleukin-12 (IL-12) production, which identify M1 macrophages, versus IL-10 production, a hallmark of M2 macrophage differentiation. We observed both TNF+ and IL-12+ cells within the IPM of CEP-MSA-immunized mice, but IL-10 staining was negative, indicating the presence of activated M1 macrophages in the pathological regions of immunized mice only (Figure 4(b)). To substantiate the IHC results, we performed mRNA quantification on IPM-infiltrating macrophages isolated by laser capture. These data confirmed that detectable levels of M1 marker genes (*IL-6*, *TNF-*α**, and *IL-1*β**) were observed only in CEP-immunized mice, whereas *IL-10* expression was not detected (Figure 4(c)). Expression of *Arg1*, another M2 marker, was not elevated in CEP-immunized mice. Interestingly, we also observed CEP-associated increased expression of *Ccl2*, a monocyte chemoattractant that has been implicated in AMD, suggesting that the Ccl2/Ccr2 axis may play a role in CEP-induced pathology. These data strongly suggest that M1 macrophages are primarily associated with the lesions we observe in the outer retina and may be the main effectors of the inflammatory response observed in this model.

### 2.5. Macrophage Recruitment into the Outer Retina of CEP-MSA-Immunized Mice Is Necessary for the Induction of Disease

**Figure 5:**
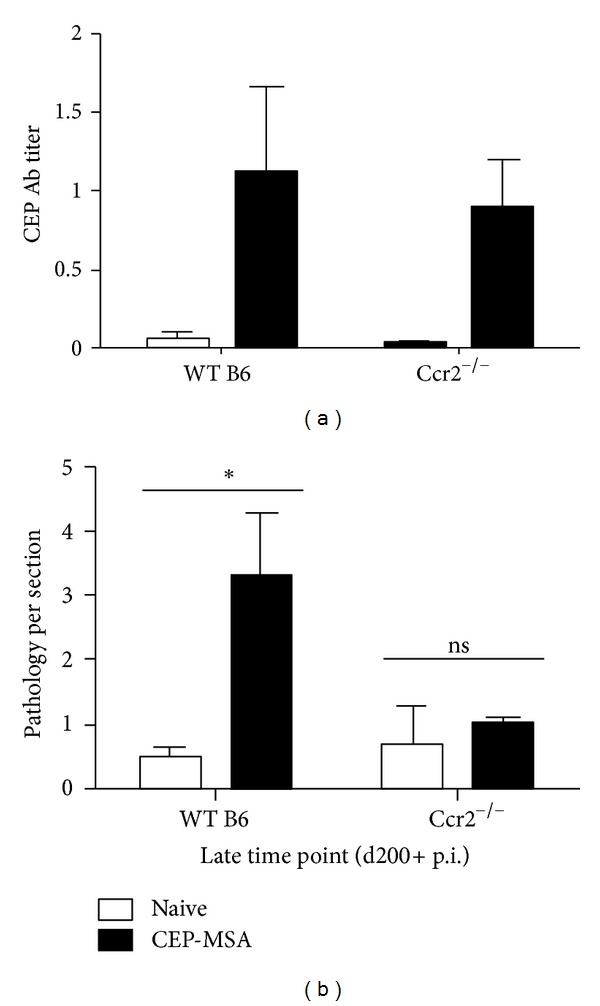
Ccr2 chemokine receptor signaling is required for macrophage recruitment to the outer retina after CEP-MSA immunization. (a) Anti-CEP antibody titers were examined following immunization of WT versus *Ccr*2^−/−^ B6 mice (*n* = 5). Naïve mice have no anti-CEP titers. Mean values are shown; error bars represent S.D. (b) Retinal pathology scores for the indicated groups at the late time point (day 200+ p.i.; *n* = 3). (*) denotes statistically significant difference (*P* < 0.05); ns: not significant. Data from one representative experiment was used for this analysis; similar results were obtained in a separate independent experiment.

 To directly test the role of macrophage recruitment into the IPM and development of outer retinal lesions we immunized Ccr2-deficient mice on the B6 background. These mice have a defect in chemokine signaling and show poor recruitment of inflammatory cells into sites of inflammation. In addition, it has been previously shown that disruption of chemokine signaling to macrophages in these Ccr2 knockout mice can have a deleterious effect on the integrity of the retina in aged mice (2 years and older) [29]. While Ccr2 deficiency did not affect the levels of anti-CEP antibody titers in young immunized mice (Figure 5(a)), *Ccr*2^−/−^ animals showed no increase of IPM-infiltrating macrophages or outer retinal lesions in immunized mice compared to those in naive age-matched controls at late recovery times (Figure 5(b)). This observation strongly suggests that macrophages, mainly recruited by Ccl2, play a causative role in the induction of tissue damage in this model.

## 3. Discussion

Immunization of mice with CEP-MSA provides a valuable model to study dry AMD from an immunological perspective, helping to dissect the immune system's role in the development of disease. Studies presented here link the AMD-like histopathological changes with the presence of macrophages in the outer retina during early stages of disease, suggesting that macrophages are involved in the underlying pathology. Notably, our data suggest that BALB/c mice tend to be more sensitive to immunization with CEP-MSA than B6 mice by having a greater magnitude and earlier significant difference of inflammatory cells in the IPM when compared to age-matched controls. In addition, dry AMD-like pathology, such as RPE cell hypertrophy, vacuolization of RPE and ROS, and RPE cell pyknosis, is also found at greater magnitude and arises earlier following immunization in the BALB/c mice. We also show that old CEP-immunized BALB/c mice develop photoreceptor cell loss. This suggests that future studies using this model would benefit from a more rapid and amplified immunopathological effect in BALB/c mice than in B6 mice, yielding results as early as 40–100 days postimmunization. 

Even if BALB/c mice show an earlier significant response to immunization with CEP-MSA than age-matched B6 mice, it is important to stress that CEP-immunized mice contain larger numbers of macrophages in the IPM and AMD-like pathology than naïve controls in both strains. Furthermore, our data showed no statistically significant differences between the two strains through time. This suggests that any age-related changes seen in immunized mice observed during the early stages of disease are of comparable magnitude regardless of strain, but that the higher number of macrophages present in the IPM of BALB/c mice makes it technically easier for quantification of disease onset. In other words, the reason that there seems to be no early differences in naïve versus CEP-MSA mice on the B6 background is because the actual number of IPM-infiltrating cells is too low at that point to achieve statistical significance. 

Differences between these two strains could also be attributed to background-specific (genetic and/or immune) mechanisms or to the reduced melanin levels in BALB/c mice. B6 mice are prone to develop T helper type 1 (Th1) responses, whereas BALB/c are Th2-prone. On the other hand, it has been shown that melanin in the RPE provides protection from light damage [30]. By showing that albino B6 (*Tyr* ^−/−^) mice have comparable inflammatory cell numbers in the IPM and AMD-like pathology with WT B6 mice in a much less robust form than BALB/c mice, the possibility that light damage largely contributes to pathology is less likely. Indeed, it has been previously shown that B6 (*Tyr* ^−/−^) are not vulnerable to light damage [31]. Therefore, we believe that at least one major reason for the observed kinetic and quantitative differences is the number of inflammatory cells in the outer retinas of BALB/c mice. Whether there are significant differences in endogenous CEP levels in the retinas of these mice, inherent differences in RPE function and/or local oxidative damage responses in the retina, or the particular contribution of specific adaptive immunity pathways, is an aspect under current investigation in our laboratory.

This work also describes in detail the differences in subretinal macrophages between these two widely used mouse strains. While many macrophage-like cells are present in the subretinal space of young naïve BALB/c mice, we have not been able to successfully identify these cells based on surface marker expression. The true nature of these baseline retinal macrophages in BALB/c mice remains unknown. Importantly, we only found subretinal CD11b+/F4/80+/CD68+ macrophages in CEP-MSA-immunized but not naive mice of either strain. A previous study has shown the presence of these macrophage-like cells in WT B6 mice but only after 20 months of age [32]. Because CEP-MSA immunization leads to the presence of these macrophages in younger mice, we believe that this is additional confirmation of the validity of our model in accelerating an endogenous aging-related process. Thus, the CEP model provides an ideal setting to study different subpopulations of retinal macrophages.

The controversial role suggested for macrophages in AMD stems primarily from the use of gene knockout mice as well as an acute model for choroidal neovascularization (CNV) that has been widely (and successfully) used to mimic wet AMD. For instance, the assertion that macrophages are antiangiogenic comes mostly from studies using laser-induced CNV [33], which is actually an acute wound healing response, not a chronic pathological state progressing from a previously established disease state, such as human wet AMD. A further complication involves the two different forms of AMD: macrophages could have different roles in dry versus wet AMD. It is important to stress that the laser-induced CNV model is a completely different system from our CEP model of dry AMD, and findings in one model will not necessarily be directly comparable to the other.

The initial evidence linking macrophages with AMD came from the analysis of mice deficient in macrophage chemokine signaling components (*Ccl*2^−/−^ and *Ccr*2^−/−^ mice) which show retinal defects similar to AMD with advanced age (2-year-old mice or older), including spontaneous CNV and “drusen” formation [29]. However, subsequent work by Luhmann et al. (2009) [32] revealed that these findings were in fact an artifact due subretinal macrophage accumulation and that any AMD-like pathology in *Ccl*2^−/−^ mice was most likely due to aging alone. An additional problem with the knockout mice mentioned previously and their use as AMD models is the fact that these strains were found to include a known mutation (rd8) that by itself results in retinal degeneration [34, 35]. Therefore most, if not all, the previously published papers using these strains must be reevaluated in that context. 

However, there is still acceptable evidence associating macrophages with AMD. For example, young macrophages inhibit CNV in the laser-induced model of wet AMD, but their antiangiogenic potential is reduced with age as they switch to an M2 phenotype [33]. More recently, it has been shown that microglia can induce RPE cells to produce proinflammatory cytokines and chemokines [36]. However, information is lacking to clarify the pathological role of macrophages at different stages of the AMD disease process, particularly at the time of onset of dry AMD before the transition to CNV. The presence of subretinal CD11b+/F4/80+/CD68+ macrophages in CEP-MSA immunized mice we show here is similarly reported in a recent paper by a different group [37]. In addition, we showed that these macrophages were M1 polarized. This suggests a strong causal link between the M1 macrophages and outer retinal lesions. 

In the original publication of our model, it was suggested that macrophages were present as a result of tissue damage and were not likely to cause disease [22]. The rationale for this conclusion was the fact that many lesions occurred in the absence of these cells. However, that original paper did not go into detail on the characterization of these cells. Missing from the first study and addressed in this paper are three key parameters that now lead to the interpretation that there is a causal relationship between M1 macrophages and dry AMD-like pathology: (i) kinetics and magnitude (quantification) of macrophage infiltration into the IPM relative to lesion development; (ii) activation status of the observed macrophages; (iii) how are the cells being recruited? This current study provides evidence for the first time that the early involvement of M1 macrophages occurs in animals that are predisposed to develop retinal lesions. We also provide the mechanism for recruitment of these cells, as Ccl2 is elevated in retinas of CEP-immunized mice, and its receptor, Ccr2, is required for macrophage infiltration into the IPM.

While we cannot completely rule out at this time that the M1 macrophages present in the IPM of CEP immunized mice are actually microglia migrating from the inner retina, it is likely that these cells come from the blood because of the systemic nature of our immunization protocol; retinal microglia are present at their normal inner retina location in Rag-deficient mice that do not develop lesions upon CEP-MSA immunization [22]. Furthermore, this model relies on the endogenous accumulation of CEP adducts in the outer retina, which should occur at equivalent rates in immunized versus naïve mice, allowing resident microglia an equal access to the CEP antigen. A more definitive distinction of the original source of these cells awaits the development of microglia-specific and/or macrophage-specific markers. Regardless, our work confirms the critical role of bone-marrow-derived macrophages in the development of retinal degeneration and provides an excellent platform to further characterize this process.

As mentioned previously, we are aware that both *Ccr*2^−/−^ and *Ccl*2^−/−^ mice develop AMD-like pathology with age [29], even though the recent work by Luhmann et al. [32, 34] has challenged this notion, at least for *Ccl*2^−/−^ mice. A major difference between these other studies and ours is that our model allows us to focus on the evaluation of relatively young animals following immunization with CEP-MSA, in contrast to the retinal lesions described previously that develop in the older knockout animals; we analyzed mice before 12 months of age, the naïve *Ccr*2^−/−^ mice develop retinal pathology after 18–20 months. Therefore, it would be difficult to make a direct comparison with our study, but it provides the opportunity to explore new mechanisms that link immunity to AMD. *Ccr*2^−/−^ mice do not lack ocular macrophages, just defective (or delayed) age-related recruitment (to the choroid). In fact, as shown by Luhmann et al. 2009 [32], “old” *Ccl*2^−/−^ mice (which closely resemble the Ccr2 macrophage phenotype) have increased macrophage recruitment to the subretinal space (the same area in which we observe macrophage infiltration in CEP-MSA mice) when compared to wild type, showing that a defective Ccl2/Ccr2 axis does not necessarily, by itself, preclude retinal infiltration of macrophages. While there is certainly a possibility that the observed pathology in CEP-immunized WT mice may not be due to macrophages, we think the *Ccr*2^−/−^ data in this paper answers that question: if macrophages did not play a detrimental role in our model (if the retinal lesions in CEP-MSA mice were macrophage independent) then we should have observed some pathology in the immunized *Ccr*2^−/−^ mice, which we did not. Because the *Ccr*2^−/−^ mice still develop CEP antibodies similar to WT (indicative of an effective adaptive immune response), the M1 phenotype of subretinal macrophages as well as the temporal relationship between macrophage infiltration and retinal lesions (macrophage recruitment precedes lesion development), we believe that our interpretation that macrophages are detrimental in our model is justified.

It is tempting to hypothesize that there could be two different populations of macrophages involved in the AMD disease process: one being early “harmful” M1 and the other being the “protective” late M2, which in turn may contribute to CNV (once disease has progressed sufficiently). We believe that our data is representative of the role of early M1 macrophages and provides a nice platform to study early events in the development of AMD. This does not exclude the idea that later cellular involvement may include M2 macrophages that could be important for resolution of disease, suggested in the published studies looking at aged Ccr2/Ccl2 knockout mice [29, 32]. In fact, it was recently shown in a retinal neuropathy injury model that IL-10-producing (M2) macrophages have a protective role [38]. The balance between M1 and M2 at different ages may actually dictate the damaged versus repaired tissue status of the retina. To support this notion, a recent paper analyzing human AMD eyes showed that AMD correlated with increased M1/M2 ratios, whereas normal aging eyes had more M2 macrophages [39]. In the context of the retina, CEP tilts the balance toward the M1 pathway for its role in inflammation-induced GA.

The enhanced presence of proinflammatory macrophages in our model offers new opportunities to investigate their role and function in AMD pathogenesis, as well as the immunological signals and inflammatory agents behind their activation and recruitment to the outer retina, a tissue historically thought of as an immunosuppressive environment. We believe that innovative immunotherapies that target the low-grade inflammatory responses at the early stages of our model can yield further promising information on the immune mechanisms that take place in response to oxidative damage in the retina. 

## 4. Conclusions

An incomplete understanding of AMD pathogenesis prevents the development of effective therapies. Current understanding of AMD recognizes oxidative stress and chronic retinal inflammation as possible causative factors. Retinal macrophages have been recognized to have a role in AMD, but their precise role (whether protective, damaging, or incidental) remains controversial. Using our AMD mouse model, we observed significant macrophage retinal infiltration that temporally preceded the onset of overt retinal pathology, suggesting a causative role for macrophages in retinal degeneration. Interestingly, mice with defective macrophage recruitment (Ccr2-deficient mice) lack macrophage retinal infiltrates and are devoid of AMD-like retinal pathology. This work uncovers an important and detrimental role for macrophages in the development of AMD. Such an understanding raises the possibility of exploring immune-modulating therapy for the treatment or prevention of retinal degeneration, especially in patients exhibiting early signs of disease. 

## 5. Materials and Methods

### 5.1. Mice

BALB/c wild-type mice, C57BL/6 wild-type mice, and *Ccr*2^−/−^ and B6(Cg)-*Tyr* ^*c*−2*J*^/J (B6-albino) mice were obtained from The Jackson Laboratory. All mice were housed in a room exposed to 300 lux (outside the cage) in a 12 hr dark/light cycle. Protocols for use of experimental animals in this study adhered to the ARVO Statement for the Use of Animals in Ophthalmic and Vision Research and were approved by the Institutional Animal Care and Use Committee of the University of Miami Miller School of Medicine. 

### 5.2. Antigen

CEP-MSA was prepared from commercially available mouse serum albumin (Sigma-Aldrich), which was converted to CEP-modified MSA following previously published procedures [40]. 

### 5.3. Immunizations

The CEP-MSA immunization protocol has been described previously [22]. In summary, mice were primed by hind leg injections of 200 *μ*g CEP-MSA in complete Freund's adjuvant (CFA; from DIFCO) at 6–10 weeks of age. At day 10 postimmunization (p.i.), the mice were challenged in the neck with 100 *μ*g CEP-MSA in incomplete Freund's adjuvant (IFA; from DIFCO), followed by a final boost with 100 *μ*g CEP-MSA in CFA in the neck seven days before harvest. Anti-CEP antibody titers at days 40–60 p.i. were quantified by ELISA as previously described [22] and used to determine efficiency of immunization. All immunized mice were compared with age-matched naïve, sham-MSA, or CFA controls. There are no significant differences among the control mice (with low to undetectable anti-CEP titers) in terms of retinal pathology and are therefore used interchangeably, depending on experimental setup. 

### 5.4. Histology

Eyes were harvested at early (40–90 days), intermediate (100–200 days), and late (over 200 days) recovery times postimmunization (p.i.). Right eyes were used for histology and were fixed in 2% paraformaldehyde and 2.5% glutaraldehyde in 0.1 M PO_4_ buffer (pH = 7.4) overnight and dehydrated in graded ethanol and propylene oxide. After polymerization in a resin mixture containing Polybed 812 (Polysciences) and Araldite 502 (Polysciences), semithin (0.7 *μ*m) sagittal sections of each eye were stained with toluidine blue and analyzed for histopathology with light microscopy using a Zeiss microscope (equipped with an AxioCam digital camera) using a 63x oil-immersion lens.

### 5.5. Quantification of Lesions and Inflammatory Cells in the IPM

Each individual mouse in this study was scored for retinal pathology on a masked fashion, using 10 sections of the right eye with at least 25–30 *μ*m intervals between each section. Scoring was divided in 2 subclasses: (1) the retinal lesion count represents the sum of RPE areas showing abnormal vesiculation, swelling, thinning, pyknosis, and cell lysis; (2) inflammatory cells were defined as dark nuclear stains of macrophage-like cells observed and counted only within the interphotoreceptor matrix compartment at the level of the photoreceptor outer segments and the apical border of the RPE. The overall pathology score for each eye is the sum of the two subclasses. The data is always presented as pathology (cells or lesions or combined) per section. 

### 5.6. Statistical Analysis of Retinal Pathology

Our data was analyzed in the Biostatistics Department at the Bascom Palmer Eye Institute with a three factor analysis of variance; the factors are: (1) strain of mice (BALB/c versus B6), (2) immunization status (naïve versus immunized), and (3) recovery time (early versus intermediate). A total of 3–5 mice were used in the analysis at each recovery time. At least two independent experiments were performed for each strain reported in this study. Repeat experiments with similar results were analyzed separately because of the use of independent batches of CEP-MSA. 

### 5.7. Immunohistochemistry

Identification of inflammatory cells in the IPM was done by immunostaining of frozen sagittal sections from the corresponding left eye for each animal. Following enucleation, eyes were embedded in OCT compound (Sakura Finetek USA), frozen on dry ice, and 8 *μ*m sections were cut using a cryostat (−20°C). Frozen sections were collected on microscope slides. The sections were fixed with 3% formaldehyde for 25 min then pretreated with a blocking solution containing 0.05% tween 20 and 3% bovine serum albumin in PBS for 1 h at room temperature to saturate nonspecific binding sites. The sections were then incubated 1 h at room temperature with the primary antibody diluted in PBS tween and 1% BSA. The following antibodies were used for surface stains: rat anti-mouse CD11b, F4/80, CD68, and CD45 (all from eBioscience). For intracellular staining, we diluted the primary antibodies in saponin buffer. The following antibodies were used for intracellular stains: rat anti-mouse TNF-*α* (BD Bioscience), IL-12p70 (Endogen), and IL-10 (BD Bioscience). Sections were then rinsed for 10 min in PBS tween and incubated with 1 : 2000 goat anti-rat Alexa Fluor 594 (Invitrogen) for 1 h at room temperature. The sections were washed three times for 10 min in PBS tween and for 10 min in PBS, coverslipped with Vectashield with DAPI for nuclear counterstaining (Vector Laboratory) and photographed in a Zeiss universal microscope (Carl Zeiss, Oberkochen, Germany) equipped for incident-light fluorescence and confocal microscopy. 

### 5.8. Laser Capture Microdissection of Outer Retina Macrophages

CFA or CEP-MSA-immunized mice were euthanized in a CO_2_ chamber, and their eyes were harvested for tissue processing. Eyes were cryoprotected in 1.5% sucrose, embedded in Tissue-Tek OCT compound (Sakura Finetek, USA), and frozen. Cryostat sections, 12 *μ*m thick, were mounted on PEN-membrane slides (Leica). Sections were then incubated in absolute ethanol and briefly stained for H&E. Single infiltrating macrophages in interphotoreceptor matrix were collected using Laser Microdissection System LMD6000 (Leica). Total RNA was extracted using the RNeasy mini kit (Qiagen) and reversely transcribed with High-Capacity cDNA Archive Kit (Applied Biosystems). cDNA was preamplified with Taqman PreAmp Master Mix Kit followed by PCR amplifications of cDNA, using Taqman probe-based gene expression assay (Applied Biosystems). Relative mRNA (in arbitrary units) was calculated using the comparative quantitation method of relative quantity (2^−ΔCt^), with actin as the calibrator for each gene of interest. Primer and probe sets were as follows: *ActB*, Mm00607939_s1, *IL-1*β**, and Mm01336189_m1; *TNF-*α**, Mm00443258_m1; *IL-6*, Mm00446190_m1; *Ccl2*, Mm00441242_m1; *IL-10*, Mm99999062_m1; *Arg1*, Mm00475988_M1.

## Supplementary Material

The Supplementary Material includes 3 figures showing another example of photoreceptor cell loss in CEP-immunized mice and accompanying inflammatory cells in the choroid and outer retina
