# Supplementary material for: Infiltration of Proinflammatory M1 Macrophages into the Outer Retina Precedes Damage in a Mouse Model of Age-Related Macular Degeneration
Source: Int J Inflam. 2013 Mar 7;2013:503725. doi: 10.1155/2013/503725 (PMC3606733; doi:10.1155/2013/503725)
Supplement: Supplementary file 1 — The Supplementary Material includes 3 figures showing another example of photoreceptor cell loss in CEP-immunized mice and accompanying inflammatory cells in the choroid and outer retina [file 503725.f1.pdf]

Supplementary Figures for:

Infiltration of Pro-Inflammatory M1 Macrophages Into the Outer Retina Precedes Damage in a Mouse Model of Age-Related Macular Degeneration

Fernando Cruz-Guilloty[a,b], Ali M. Saeed[a], Jose J. Echegaray[a], Stephanie Duffort[a], Asha Ballmick[a], Yaohong Tan[a], Michel Betancourt[a], Eduardo Viteri[a], Ghansham C. Ramkhellawan[a], Eric Ewald[a], William Feuer[a], DeQiang Huang[a], Rong Wen[a], Li Hong[c], Hua Wang[c], James M. Laird[c], Abdoulaye Sene[d], Rajendra S. Apte[d], Robert G. Salomon[c], Joe G. Hollyfield[e] and Victor L. Perez[a,b]

[a]Bascom Palmer Eye Institute, Department of Ophthalmology, University of Miami Miller School of Medicine, Miami, FL, USA 33136

[b]Department of Microbiology and Immunology, University of Miami Miller School of Medicine, Miami, FL, USA 33136

[c]Department of Chemistry, Case Western Reserve University, Cleveland, OH, USA 44106

[d]Department of Ophthalmology and Visual Sciences, Washington University School of Medicine, St. Louis, Missouri, USA. 63110

[e]Cole Eye Institute, Department of Ophthalmology, Cleveland Clinic Lerner College of Medicine, Cleveland, OH, USA 44195

Corresponding author: Victor L. Perez, Associate Professor, Ophthalmology, Immunology & Microbiology, University of Miami Miller School of Medicine, Bascom Palmer Eye Institute, Miami, FL, USA 33136. Vperez4@med.miami.edu

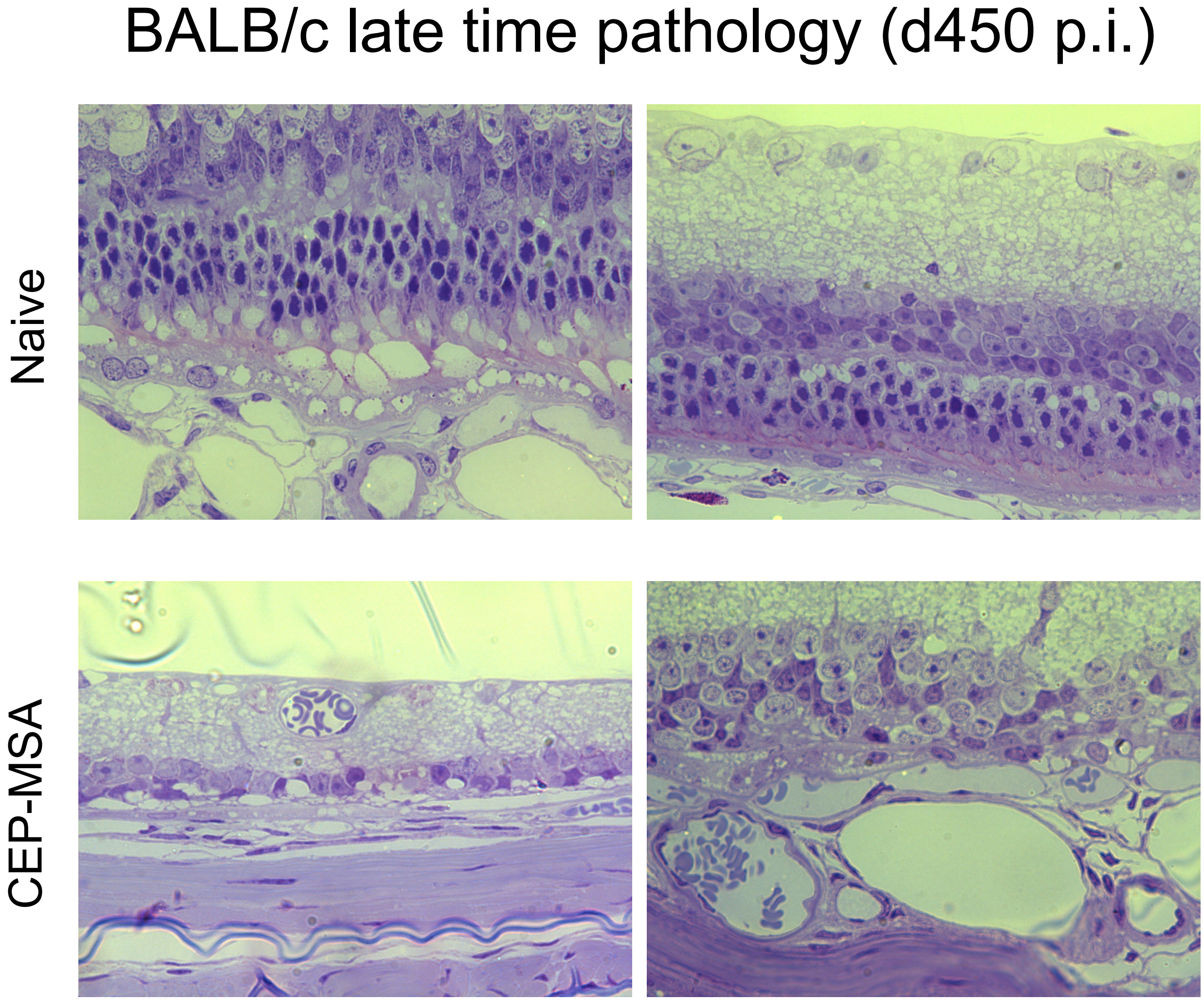

Suppl. Fig. 1. *Age-related retinal degeneration in BALB/c mice is exacerbated by CEP-MSA immunization.* Naïve BALB/c mice present obvious signs of outer retinal degeneration by ~18 months of age, such as reduced density of photoreceptor cell nuclei and thinning of the rod outer segment. However, pathology in age-matched CEP-MSA-immunized mice is more severe, with signs of geographic atrophy, areas of complete loss of the photoreceptor cells and possible signs of choroidal neovascularization. Representative images from 3-5 mice per group are shown.

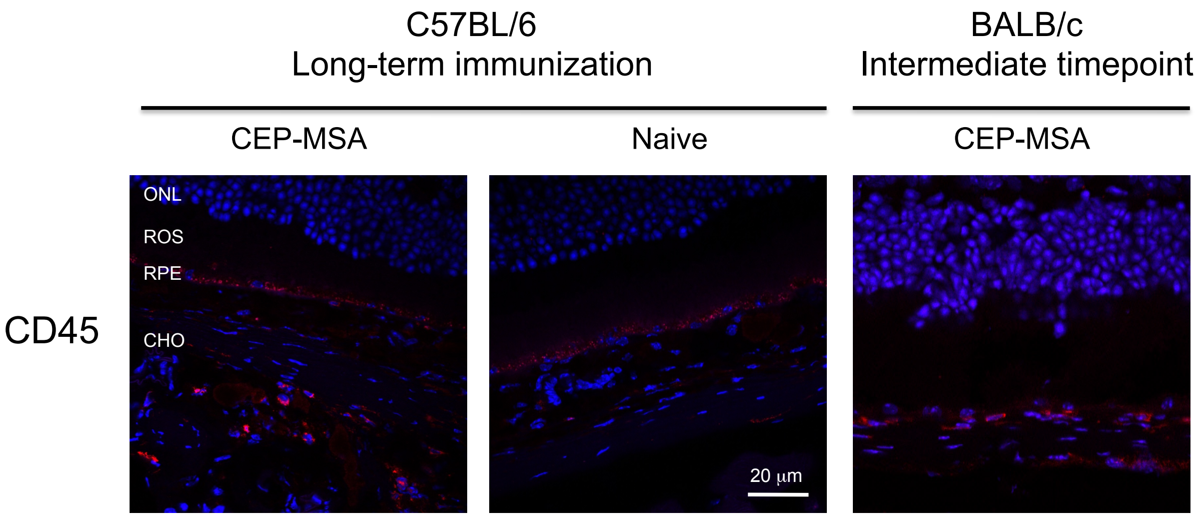

Suppl. Fig. 2. *Inflammatory cells surround the retinas of CEP-MSA immunized mice*. Anti-CD45 stains of frozen sections show the presence of increased numbers of inflammatory cells in the choroid of immunized mice in the B6 background, relative to age-matched naïve mice. Similar data is observed in the BALB/c mice, even at earlier time-points. INL, inner nuclear layer; ONL, outer nuclear layer; ROS, rod outer segment; RPE, retinal pigment epithelium; CHO, choroid. The scale marker represents a 20 μm length.

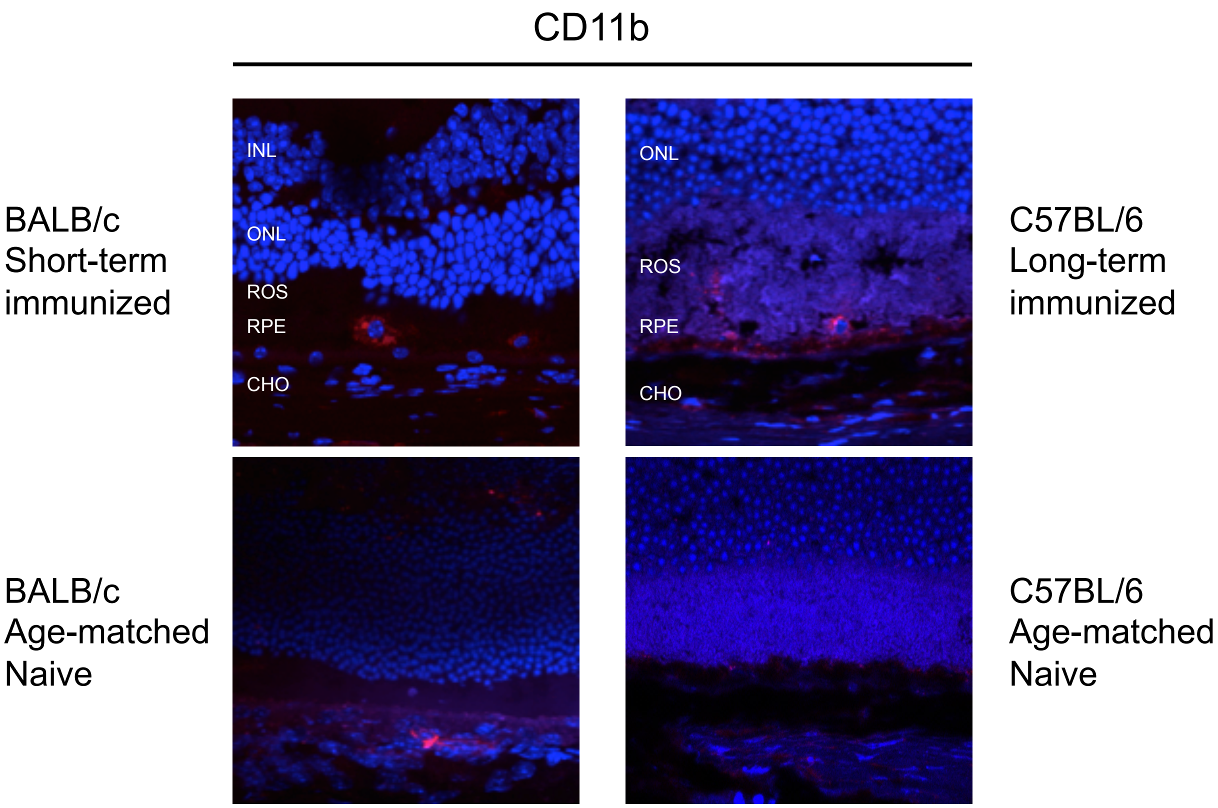

Suppl. Fig. 3. *Retinal CD11b+ macrophages in CEP-MSA-immunized mice.* Anti-CD11b was used to stain frozen sections of eyes from CEP-MSA immunized BALB/c mice at d40-100 p.i., C57BL/6 mice at d200+ p.i. or age-matched naïve controls. Even though naïve mice show macrophage-like cells in the ROS/RPE region by histology, these cells are not CD11b positive. On the other hand, CEP-MSA-immunized mice contain CD11b+ cells in the relevant regions, with BALB/c mice showing infiltration at early time points. Representative images from 3-5 mice per group are shown. INL, inner nuclear layer; ONL, outer nuclear layer; ROS, rod outer segment; RPE, retinal pigment epithelium; CHO, choroid.
